# Leukoencephalopathy with accumulated succinate is indicative of *SDHAF1* related complex II deficiency

**DOI:** 10.1186/1750-1172-7-69

**Published:** 2012-09-20

**Authors:** Andreas Ohlenbusch, Simon Edvardson, Johannes Skorpen, Alf Bjornstad, Ann Saada, Orly Elpeleg, Jutta Gärtner, Knut Brockmann

**Affiliations:** 1 Department of Pediatrics and Pediatric Neurology, Georg August University, Robert Koch Str. 40, Göttingen, 37075, Germany; 2 Pediatric Neurology Unit, Hadassah, Hebrew University Medical Center, Jerusalem, Israel; 3 Department of Pediatric Medicine, Child Habilitation Unit, Ålesund Hospital, Ålesund, Norway; 4 Department of Pediatrics, Drammen Sykehus, Drammen, Norway; 5 The Department of Genetic and Metabolic Diseases, Hadassah, Hebrew University Medical Center, Jerusalem, Israel

**Keywords:** Succinate dehydrogenase, Leukoencephalopathy, SDHAF1, Leigh syndrome, Complex II deficiency, Assembly factor

## Abstract

**Background:**

Deficiency of complex II (succinate dehydrogenase, SDH) represents a rare cause of mitochondrial disease and is associated with a wide range of clinical symptoms. Recently, mutations of *SDHAF1,* the gene encoding for the SDH assembly factor 1, were reported in SDH-defective infantile leukoencephalopathy. Our goal was to identify *SDHAF1* mutations in further patients and to delineate the clinical phenotype.

**Methods:**

In a retrospective data collection study we identified nine children with biochemically proven complex II deficiency among our cohorts of patients with mitochondrial disorders. The cohort comprised five patients from three families affected by SDH-defective infantile leukoencephalopathy with accumulation of succinate in disordered cerebral white matter, as detected by *in vivo* proton MR spectroscopy. One of these patients had neuropathological features of Leigh syndrome. Four further unrelated patients of the cohort showed diverse clinical phenotypes without leukoencephalopathy. *SDHAF1* was sequenced in all nine patients.

**Results:**

Homozygous mutations of *SDHAF1* were detected in all five patients affected by leukoencephalopathy with accumulated succinate, but not in any of the four patients with other, diverse clinical phenotypes. Two sisters had a mutation reported previously, in three patients two novel mutations were found.

**Conclusion:**

Leukoencephalopathy with accumulated succinate is a key symptom of defective complex II assembly due to *SDHAF1* mutations.

## Background

Deficiency of complex II (succinate dehydrogenase, SDH) is a rare cause of disordered oxidative phosphorylation (OXPHOS) and is associated with a wide range of clinical symptoms. Among our cohorts of more than 1200 patients with defects in OXPHOS we found only nine with biochemically proven deficiency of complex II.

In 2001 we reported a characteristic finding in localized *in vivo* cerebral proton magnetic resonance spectroscopy (MRS) in three patients from two unrelated families, two German sisters of Turkish origin (family A) and one Norwegian boy (family B), presenting with symptoms and MRI signs of leukoencephalopathy [1]. MRS revealed a prominent singlet at 2.40 ppm in cerebral and cerebellar white matter originating from accumulated succinate in affected white matter. Biochemical investigations demonstrated isolated deficiency of complex II in muscle and fibroblasts of these patients.

Recently, homozygous mutations in *SDHAF1*, encoding a new LYR-motif protein, were detected in 2 families from Turkey and Italy with several children affected by infantile leukoencephalopathy with defective SDH [2,3].

We investigated whether *SDHAF1* is mutated in our nine patients with complex II deficiency with either SDH-defective leukoencephalopathy or other, diverse clinical phenotypes without leukoencephalopathy.

## Patients and methods

The Table 1 summarizes the clinical, neuroradiological, and biochemical features of five patients from three families with SDH-defective leukoencephalopathy and 4 unrelated patients with other clinical phenotypes of complex II deficiency. Details of clinical and neuroradiological features of patients #1, #2 and #3 were described previously [1]. 

**Table 1:** Clinical, neuroradiological, biochemical and genetic features of five patients from three families with SDH-defective leukoencephalopathy and four unrelated patients with other, diverse phenotypes of complex II deficiency

**Patient (origin)**	**Sex**	**Present age**	**Affected/unaffected siblings**	**Consanguinity of parents**	**Age at onset**	**Presenting sign**	**MRI, proton MRS of the brain**	**Postmortem**	**Complex II activity measured in: (SDH residual activity normalized to CS)**	***SDHAF1*mutation**
1 (T)	f	Died at 18 mo	1 (#2)/2	+	10 mo	Motor regression	Bilateral LE, succinate peak	Leigh syndrome	Muscle (46%), fibroblasts (24%)	c.164 G > C ^a^ p.Arg55Pro

2 (T)	f	Died at 11 yrs	1 (#1)/2	+	10 mo	Motor regression	Bilateral LE, succinate peak	-	Fibroblasts (74%)	c.164 G > C ^a^ p.Arg55Pro

3 (N)	m	16 yrs	-/2	Uncertain	20 mo	Spasticity, clumsiness	Bilateral LE, succinate peak	-	Fibroblasts (16%)	c.22C > T ^b^ p.Gln8X

4 (P)	f	Died at 5 yrs	1 (#5)/1	+	14 mo	Spasticity, motor regression	Bilateral LE, succinate peak	-	Lymphocytes (39%)	c.170 G > A ^b^ p.G57E

5 (P)	f	3 yrs	1 (#4)/1	+	4 mo	Spasticity	Bilateral LE, succinate peak	-	Lymphocytes (58%)	c.170 G > A ^b^ p.G57E

6 (G)	m	15 yrs	-/-	-	3 yrs	Exercise intolerance	Normal, no succinate peak	-	Muscle (55%)	None
7 (J)	f	Died at 2 yrs	-/-	-	18 mo	Acute liver failure, liver transplantation	-	-	Liver	None
									(42%)	
8 (J)	f	9 yrs	-/2	-	birth	Psychomotor retardation, muscle weakness, hearing loss	Normal	-	Muscle	None
									(45%)	
9 (J)	f	7 yrs	-/-	-	birth	Psychomotor retardation, muscle weakness, hypotonia	Normal	-	Muscle	None
									(71%)	

*SDH* succinate dehydrogenase; *CS* citrate synthase; *(T)* Turkish origin; *(N)* Norwegian origin; *(P)* Palestinian origin; *(G)* German origin; *(J)* Jewish origin; *f* female; *m* male; *mo* months; *yrs* years; + = present; - = absent/not done; LE = leukoencephalopathy; ^a^ mutation reported previously [2], ^b^ mutation not reported previously.

In family A, patients #1 and #2 were the second and fifth children of healthy, consanguineous (first cousins) German parents of Turkish origin belonging to a big pedigree where *SDHAF1* mutations were previously described [2]. Both sisters presented with motor deterioration and spasticity in the 2^nd^ half of their first year of life. MRI of the brain revealed extensive T2-hyperintensities in cerebral and cerebellar white matter, and cerebral proton MRS demonstrated accumulation of succinate. The elder sister died from multiorgan failure with severe lactic acidosis at age 18 months. Postmortem examination revealed histopathological features and topic patterns of a multifocal spongiform encephalomyelopathy consistent with Leigh syndrome [1]. The younger sister showed severe motor disability with marked spastic tetraparesis and relatively preserved cognitive abilities. She died at age 11 years.

**Figure 1:**
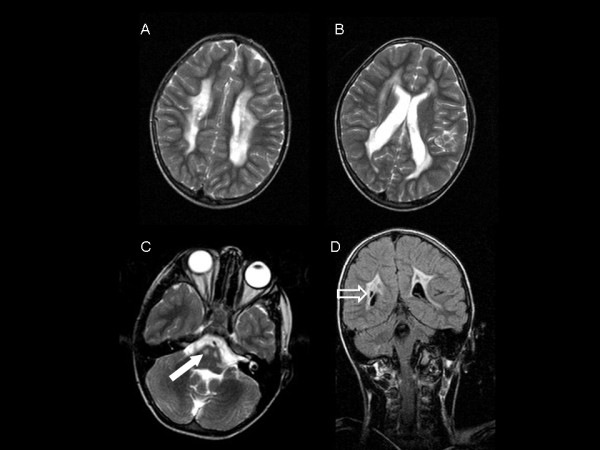
**(A-C) Axial T2-weighted and (D) coronal FLAIR-weighted MR images of patient 3 at 9 years of age show widespread bilateral T2-hyperintensities in cerebral periventricular white matter.** Involvement of the pons (**C**, full white arrow) and cystic lesions (**D**, open arrow) are visible. Peripheral U-fibers are spared. Lesions are largely unchanged compared to neuroimaging at age 4 years^1^.

In family B, patient #3 was the first of three children of allegedly unrelated Norwegian parents coming from neighboring areas. With onset at 20 months increasing spasticity and clumsiness were observed. Cerebral MRI and proton MRS indicated a leukoencephalopathy with accumulation of succinate. Biochemical analysis of fibroblasts demonstrated an isolated deficiency of complex II. At present, at age 16 years, his main clinical feature is spastic paraplegia. He has suffered single epileptic seizures but has no preventive treatment. The cognitive function is tested to be within normal range. His fine motor skills and language function are good, and he is attending ordinary school with some facilitation. A follow-up cranial MRI performed at age 9 years showed leukoencephalopathy with supratentorial bilateral T2-hyperintensities (Figure 1), largely unchanged compared with neuroimaging performed 5 years before [1]. 

In family C, patients #4 and #5 were the first and second of three daughters of consanguineous Palestinian parents. With onset at 14 and 4 months, respectively, they showed motor regression and spasticity. In patient #4, best motor function was standing up, best mental function was speaking a few words. In patient #5, best motor function was head control at 5 months, best social function was smiling at 3 months. In both girls MRI and proton MRS of the brain revealed bilateral leukoencephalopathy and accumulation of succinate. Complex II deficiency was demonstrated in muscle or lymphocytes or both. Patient #4 died at age 5 years from pneumonia. Postmortem was not performed.

Additional four patients (#6 to #9) presented with other, diverse clinical features. Patient #6 showed exercise intolerance and SDH-defective myopathy. He had normal cognitive abilities with very good performance at school as well as normal MRI and MRS of the brain. After initial normal development, patient #7 suffered from acute liver failure requiring liver transplantation with fatal outcome at age 2 years. Psychomotor retardation with muscle weakness and hearing impairment were main clinical features of patient #8. Patient #9 showed psychomotor retardation as well as muscular hypotonia and weakness. In all of these four patients, complex II deficiency was demonstrated biochemically in muscle or liver. MRI of the brain was performed in patients #6, #8, and #9 (with additional proton MRS of the brain in #6) and did not reveal leukoencephalopathy or any other abnormalities.

### Genetic analysis

Total genomic DNA was extracted from peripheral blood leukocytes by standard techniques. Primers for DNA amplification and sequencing were designed to cover exon 1 of *SDHAF1* along with flanking segments (GenBank Reference No. NC_000019.9). Screening for sequence variants was performed using the BigDye^TM^ Terminator Ready Reaction chemistry on an ABI PRISM 3100 Avant genetic analyzer (Applied Biosystems, Darmstadt, Germany). All identified mutations were confirmed by direct sequencing of two different PCR amplification products on forward and reverse strands. Primer sequences as well as PCR and sequencing conditions are available on request. Informed consent was obtained for each patient from the parents. The Institutional Review Board approved the study.

## Results

Patients #1 and #2, siblings of Turkish origin, were homozygous for a missense mutation c.164 G > C, corresponding to p.Arg55Pro, with both unaffected parents being heterozygous for the mutation.

We found a homozygous c.22C > T nonsense mutation in patient #3 of Norwegian origin predicted to result in a premature stop of translation (p.Gln8X). Two healthy siblings as well as the unaffected parents were heterozygous carrier for this variant.

In patients #4 and #5, siblings from consanguineous parents of Palestinian origin, a missense mutation c.170 G > A, corresponding to p.Gly57Glu was detected. This mutation affects the same highly conserved residue, Gly57, as the mutation Gly57Arg reported previously in an Italian family [2]. This observation indicates a fundamental role for Gly57 for the function of SDHAF1.

Molecular analysis of the coding sequence as well as of adjacent promoter and 3′UT regions revealed no sequence alterations in patients #6 to #9.

## Discussion

Mutation analysis of the *SDHAF1* gene revealed mutations in five patients with SDH-defective infantile leukoencephalopathy. The missense mutation detected in patients #1 and #2 was reported previously in a large multiconsanguineous kindred of Turkish origin with several affected children [2,3]. Family history of the siblings described here (patients #1 and #2) indicates common ancestry with those patients. The homozygous nonsense mutation demonstrated in patient #3 and the homozygous missense mutation detected in patients #4 and #5 were not reported before.

Succinate dehydrogenase participates in the electron transfer in the respiratory chain and in succinate catabolism in the Krebs cycle and consists of four subunits, all encoded by the nuclear genome [4]. Isolated complex II deficiency is a relatively rare cause of mitochondrial disease compared to other respiratory chain defects, but is associated with a wide range of clinical features [5]. Mutations in the four genes, *SDH-A, -B, -C, -D*, have been reported, with remarkably diverse phenotypes. Mutations in the *SDHA* gene were found to be associated with Leigh syndrome [6], late onset neurodegenerative disease [7] and dilated cardiomyopathy [8]. Heterozygous germline mutations in *SDHA, SDHB, SDHC,* and *SDHD* cause hereditary paragangliomas and pheochromocytomas [9], and germline mutations in *SDHB* and *SDHC* were found to be associated with gastrointestinal stromal tumors [4]. Recently, a mitochondrial encephalopathy was reported to be caused by *SDHD* mutations [10].

Whereas an increasing number of assembly factors have been identified for complex I, III, and cytochrome oxidase, little was known concerning the assembly of complex II. Recently two genes involved in this process were detected in humans, and the first, termed *SDHAF1*, was found by linkage analysis in two families with SDH-defective infantile leukoencephalopathy [2]. Yeast experimentation indicated that the protein encoded by this gene is required for the stable assembly and full function of the SDH complex. The protein was thus termed SDH assembly factor 1 (SDHAF1) [2].

In the same year, mutations in the *SDHAF2 (SDH5)* gene encoding a protein necessary for the flavination of the subunit SDHA were detected in patients with paraganglioma [10]. The cause of such diverse phenotypes associated with defective assembly factors of complex II remains enigmatic to date.

Our results confirm the pathogenicity of *SDHAF1* mutations in infantile leukoencephalopathy due to defective succinate dehydrogenase. Our patients with complex II deficiency not associated with leukoencephalopathy but with other, diverse clinical phenotypes including myopathy with exercise intolerance, acute liver failure, psychomotor delay, muscle weakness, and hearing impairment did not carry a *SDHAF1* mutation. Further studies will clarify whether infantile leukoencephalopathy with accumulation of succinate, readily detectable by *in vivo* proton MR spectroscopy of the brain, is pathognomonic for SDHAF1 deficiency.

To date, clinical features comprising motor regression with spasticity and neuroradiological features including bilateral leukoencephalopathy with elevated succinate on cerebral proton MRS are the suggestive findings pointing to a *SDHAF1* mutation.

Treatment with riboflavin was found to be effective in selected mitochondrial disorders, including SDH deficiency [3]. In our cohort, riboflavin treatment was applied in patients #2, #4, and #5 (*SDHAF1* mutations) as well as #6 (SDH-defective myopathy with exercise intolerance). Riboflavin treatment resulted in no discernable effect in the 3 patients with *SDHAF1* mutations. Patient #6 had clear benefit from this treatment with markedly prolonged motor endurance.

The clinical course in our five patients with *SDHAF1* mutations is strikingly diverse. Patients #1, #2, and #4, all carrying missense mutations, died at age 18 months, 11 years, and 5 years, respectively, with histopathological features of Leigh syndrome in one of them. In contrast, patient #3, who carries a stop mutation, follows a milder course with spastic paraparesis as the main clinical feature 14 years after onset and stable white matter changes on MRI over many years. We hypothesize that an atypical starting codon could be present resulting in synthesis of a partially functional protein. Further studies are needed to elucidate the pathomechanism of this stop mutation. This variation points to influential further genetic or epigenetic factors shaping the phenotype of defective complex II assembly due to mutated *SDHAF1*.

## Abbreviations

SDH: Succinate dehydrogenase; SDHAF1: SDH assembly factor 1; OXPHOS: Oxidative phosphorylation; MRI: Magnetic resonance imaging; MRS: Magnetic resonance spectroscopy; 3′UT region: 3′ untranslated region; LYR-motif: Pattern in protein structure consisting of leucine, tyrosine, and arginine.

## Competing interests

The authors declare that they have no competing interests.

## Authors’ contributions

AO carried out the molecular genetic studies. SE, AB, JS contributed with clinical data. AS carried out the biochemical investigations. OE contributed with clinical and genetic information and interpretation of data. JG participated in the design of the study and interpretation of data. KB conceived of the study, provided clinical information and drafted the manuscript. All authors participated in finalizing the manuscript and read and approved the final manuscript.
